# Curcumin induces G2/M cell cycle arrest and apoptosis of head and neck squamous cell carcinoma *in vitro* and *in vivo* through ATM/Chk2/p53-dependent pathway

**DOI:** 10.18632/oncotarget.17096

**Published:** 2017-04-13

**Authors:** An Hu, Jing-Juan Huang, Jing-Fei Zhang, Wei-Jun Dai, Rui-Lin Li, Zhao-Yang Lu, Jun-Li Duan, Ji-Ping Li, Xiao-Ping Chen, Jing-Ping Fan, Wei-Hua Xu, Hong-Liang Zheng

**Affiliations:** ^1^ Department of Otolaryngology, Gongli Hospital, Second Military Medical University, Pudong New Area, Shanghai, 200135, China; ^2^ Department of Cardiology, Shanghai Chest Hospital, Shanghai Jiaotong University, Shanghai, 200030, China; ^3^ Department of Gerontology, Xinhua Hospital, Shanghai Jiaotong University School of Medicine, Shanghai, 200092, China; ^4^ Department of Otolaryngology, Renji Hospital, Shanghai Jiaotong University School of Medicine, Pudong New Area, Shanghai, 200127, China; ^5^ Department of Otolaryngology-Head and Neck Surgery, Changzheng Hospital, Second Military Medical University, Shanghai, 200003, China; ^6^ Department of Otolaryngology-Head and Neck Surgery, Changhai Hospital, Second Military Medical University, Shanghai, 200433, China

**Keywords:** cell cycle arrest, apoptosis, ATM/Chk2/p53 signal pathway, head and neck squamous cell carcinoma, curcumin

## Abstract

Studies have demonstrated that curcumin (CUR) exerts its tumor suppressor function in a variety of human cancers including head and neck squamous cell carcinoma (HNSCC). However, the exact underlying molecular mechanisms remain obscure. Here, we aim to test whether CUR affects ATM/Chk2/p53 signaling pathway, leading to the induction of cell cycle arrest, inhibition of angiogenesis of HNSCC *in vitro* and *in vivo*. To this end, we conducted multiple methods such as MTT assay, Invasion assay, Flow cytometry, Western blotting, RT-PCR, and transfection to explore the functions and molecular insights of CUR in HNSCC. We observed that CUR significantly induced apoptosis and cell cycle arrest, inhibited angiogenesis in HNSCC. Mechanistically, we demonstrated that CUR markedly up-regulated ATM expression and subsequently down-regulated HIF-1α expression. Blockage of ATM production totally reversed CUR induced cell cycle arrest as well as anti-angiogenesis in HNSCC. Moreover, our results demonstrated that CUR exerts its antitumor activity through targeting ATM/Chk2/p53 signal pathway. In addition, the results of xenograft experiments in mice were highly consistent with *in vitro* studies. Collectively, our findings suggest that targeting ATM/Chk2/p53 signal pathway by CUR could be a promising therapeutic approach for HNSCC prevention and therapy.

## INTRODUCTION

Head and neck squamous cell carcinoma affects 600,000 people worldwide annually, including 42,000 in the United States [1]. Although remarkable progresses in treatment modalities in the past few years, the survival rate of HNSCC is less than satisfactory [2]. Close to half of these (30 to 50%) patients develop local recurrence and another 10–40% of patients develop distant metastasis due to field cancerization [3, 4]. Therefore, identifying more effective agents to improve therapeutic intervention for HNSCC patients and better understanding the potential molecular mechanisms of this disease are crucial [5].

Preservation of genomic integrity is a vital process for cell homeostasis [6]. Indeed, failure to accurately repair DNA damage has been correlated with numerous pathologies including cancer [6, 7]. In response to DNA damage, the ataxia-telangiectasia mutated (ATM) is activated through autophosphorylation of the Ser1981 residue and activates the distal transducer kinase checkpoint kinase 2 (Chk2), resulting in G2/M checkpoint initiation [6]. Others have reported that p53 was identified as important mediator of angiogenesis [8]. Evidence also supported that p53 up-regulates murine double minute 2-induced ubiquitination of the HIF-1α [9]. Therefore, we tried to explore the potential molecular mechanisms of ATM/Chk2/p53 signal pathway as a mediator of G2/M arrest and angiogenesis of HNSCC.

Curcumin (CUR) is the primary active ingredient of turmeric, which has been used in Chinese medicine over the years [10]. Accumulating evidences suggest that CUR shows its anti-tumor activity by modulating various targets either through direct interaction or through modulation of gene expression [11, 12]. Moreover, CUR has been demonstrated to possess direct antiangiogenic activity *in vivo* [13–15]. Further study showed that CUR exerted its antitumor activity involved in reactivation of receptor activator of NF-κB and inactivation of STAT3 in glioblastoma cells [16]. Additionally, CUR was discovered to suppress the cell growth through inhibition of histone deacetylase 4 (HADC4) and NF-κB pathways in medulloblastoma cells [17, 18]. Although many investigations have demonstrated the molecular basis of CUR-mediated cell growth inhibition, the underlying role has not been fully elucidated. Based on the above considerations, the objective of this present investigation was to explore the effect of CUR on ATM/Chk2/p53 signaling pathway in HNSCC.

## RESULTS

### CUR inhibited proliferation of HNSCC cells *in vitro*

**Figure 1:**
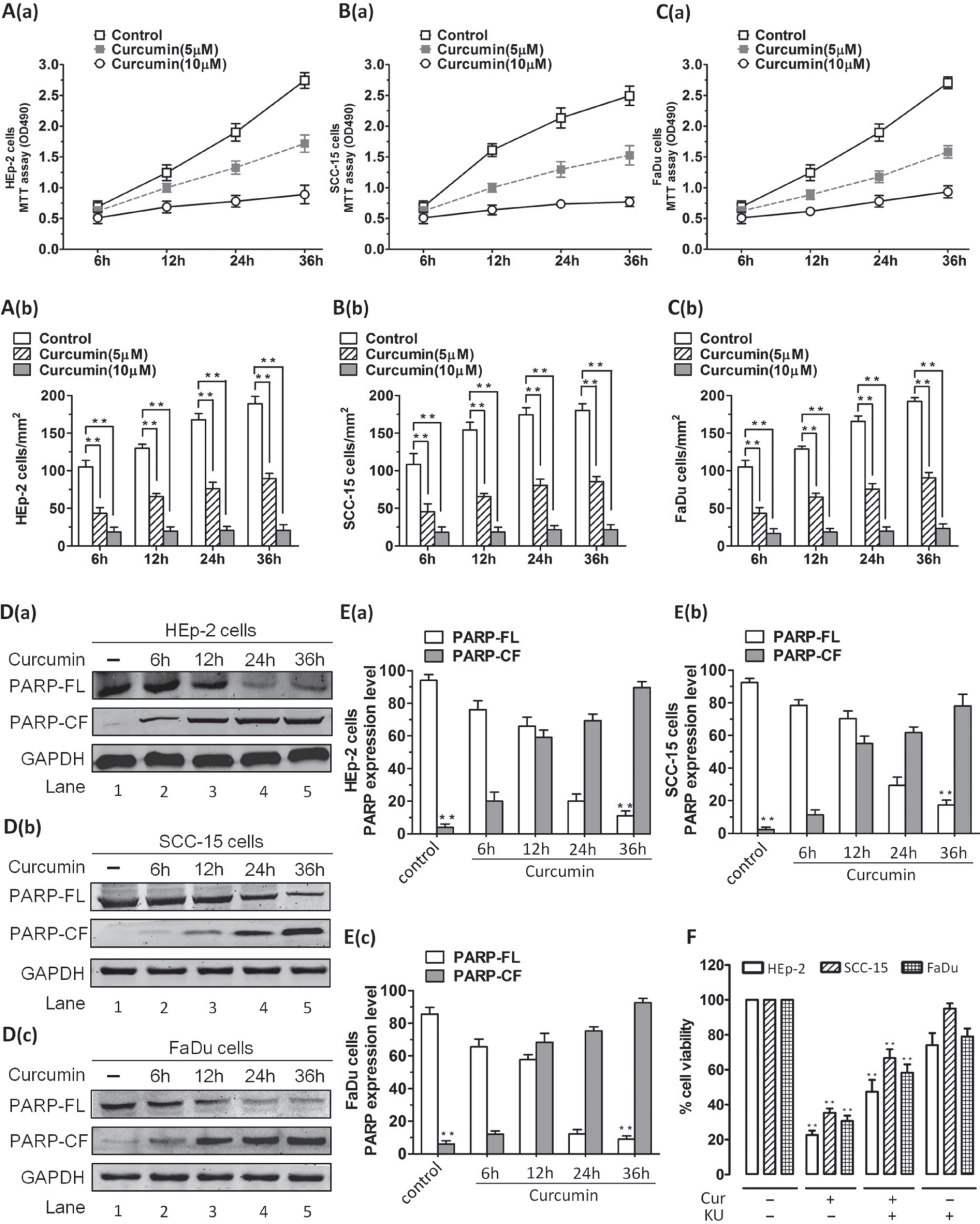
Effect of CUR on the proliferation of HNSCC cells *in vitro* HNSCC cells were treated with different concentrations of CUR for 36 h. The effect of CUR on the proliferation of these cells was analyzed by MTT assay. (**A**–**C**) Cell viability assays measuring growth and proliferation were performed in HEp-2, SCC-15, FaDu cells. A significant concentration-dependent decrease in viability of all cell lines was noted in response to treatment with CUR. 10 μM CUR inhibited cell lines growth at rates about 55–75% to those of control cells (*P* < 0.01; *n* = 3). (**D**–**E**) Cell lines were treated with CUR (10 μM) and harvested. Then whole cell lysates were subjected to Western Blot analysis with anti-PARP, or anti-GAPDH. Moreover, treatment of cell lines with CUR triggered a marked increase in proteolytic cleavage of PARP (*P* < 0.01; *n* = 3). (**F**) After treatment with 10 μM KU55933, the apoptosis rate was significant reduced in cell lines. Blots shown are representative of three independent experiments. Data were expressed as mean ± SD. **P < 0.01, versus control group. (FL, full length; CF, cleaved fragment).

To address the probable anticancer mechanism of CUR via ATM/Chk2/p53-dependent signal pathway *in vitro*, we first evaluated cell proliferation in cell lines. Human HNSCC cell lines (HEp-2, SCC-15, FaDu cells) were cultured with 5 μM or 10 μM of CUR for 36 hours, then analyzed for the growth inhibitory effects using MTT assay. 10 μM CUR inhibited cell lines proliferation at rates of 55–75% to those untreated cells (Figure 1A–1C; *P* < 0.01; *n* = 3). The expression level of proteolytic cleavage of PARP was obviously up-regulated in CUR-treated cell lines (Figure 1D–1E; *P* < 0.01; *n* = 3). Moreover, KU55933(a specific inhibitor of ATM) partially reversed the CUR mediated cell viability attenuation. After treatment with 10 μM KU55933, the apoptosis rates of cell lines were dramatically decreased (Figure 1F).

### CUR caused apoptosis of HNSCC and induced activation of ATM mediated signaling pathways

**Figure 2:**
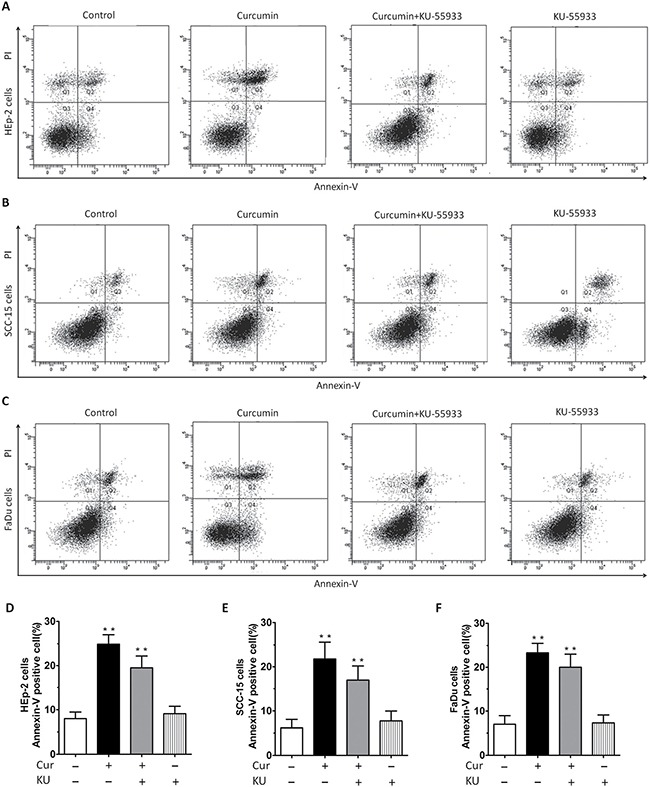
Annexin-V/PI staining showed that CUR treatment induced apparent apoptosis in HEp-2, SCC-15, FaDu cells (**A**–**C**) Annexin-V/PI staining showed that 10 μM CUR treatment for 36 h induced apparent apoptosis in cell lines. (**D**–**F**) After treatment with CUR, the cells lines showed apoptosis at rates of 17.20%, 19.54% and 21.60%, respectively. Cells lines were treated with CUR in the presence or absence of the ATM inhibitor KU55933 for 24 h, followed by assessment for cell viability using MTT assays. After treatment with KU55933, the apoptosis rate was significantly reduced, not only in HEp-2 cells group, but also in SCC-15 and FaDu cells group.

Results of HNSCC proliferation assay also indicated that CUR not only inhibited cells proliferation, but also caused cells step into death. In order to clarify apoptosis or necrosis, we conducted Annexin V-FITC/PI assay and the results determined by flow cytometry showed that percent of apoptosis cells of HNSCC was increased apparently after treatment with CUR (Figure 2A–2F), indicating that CUR has the ability to induce apoptosis of HNSCC. Annexin V-FITC/PI assay indicated that 10 μM CUR treatment for 36 h induced significant apoptosis in cell lines. As shown in Figure 2A–2C, after treatment with CUR (10 μM), the cells lines showed apoptosis rate about 17.20%, 19.54% and 21.60%. And after exposure to 10 μM KU55933, three HNSCCs (HEp-2, SCC-15, FaDu cells) had similar lower levels of apoptosis rate than the control cells (Figure 2D–2F).

### CUR mediated DNA damage caused G2/M arrest and targeted ATM mediated signaling pathways

**Figure 3:**
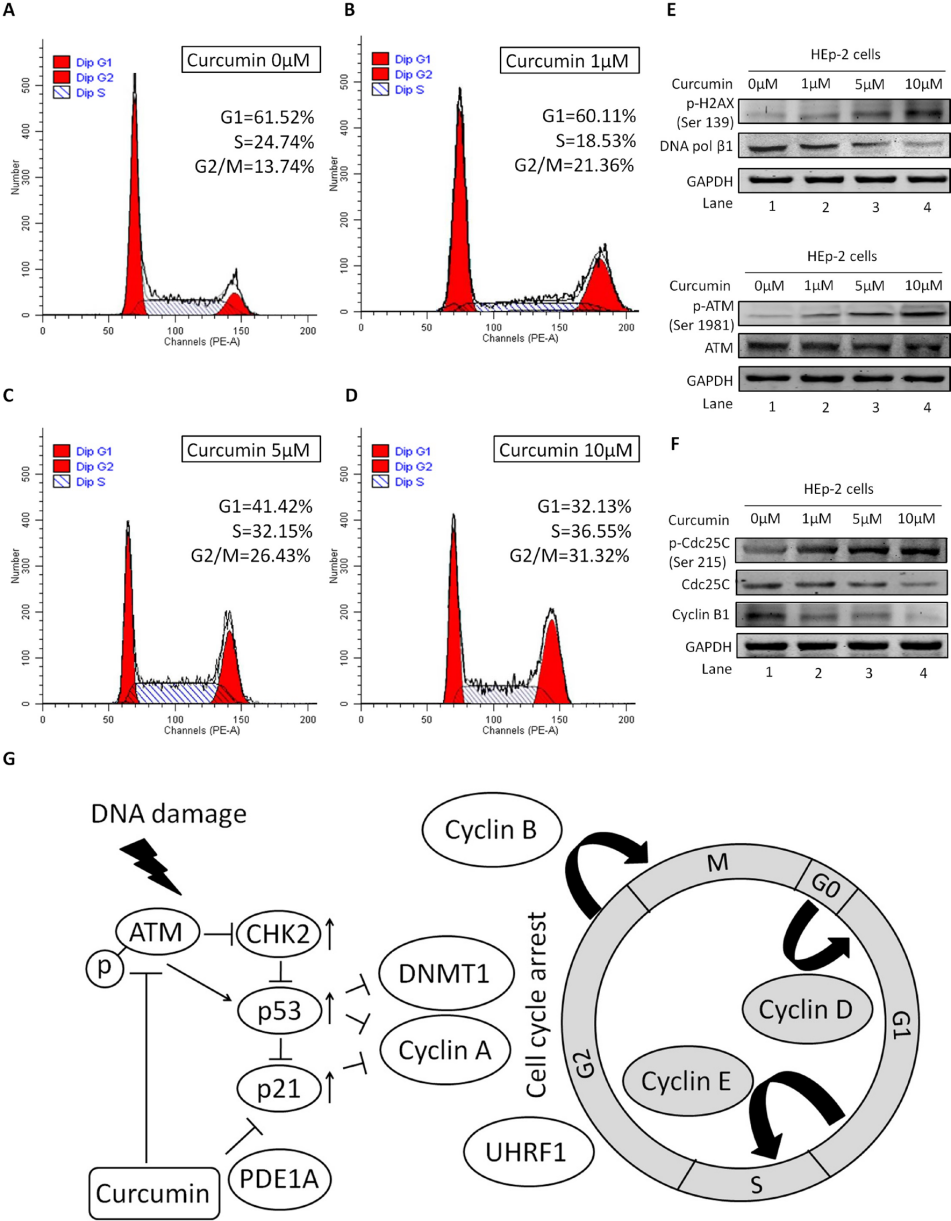
Role of ATM/Chk2/p53 in CUR mediated G2/M cell cycle arrest HEp-2 cells were treated with or without CUR for 24 h. Cells were collected and analysed for cell cycle distribution by flow cytometry. (**A**–**D**) CUR increased the proportion of cells in G2/M phase and decreased that in the G1 phase, compared with the control group (*P < 0.01). FCM analysis showed CUR arrested HEp-2 cell cycling in the G2/M phase. (**E**) We observed an increased phosphorylation of H2A.X at Ser-139. At the same time, our results show that CUR treatment decreased the expression of DNA polymerase β1. (**F**) Treatment of HEp-2 cells with CUR increased the phosphorylation of ATM at Ser-1981. On the other hand, substantial phosphorylation of Cdc25C at Ser-216 was observed in HEp-2 cells treated with CUR. Exposure of HEp-2 cells with 10 μM CUR for 24 h significantly reduced the expression of Cyclin B1. (**G**) CUR causes cell cycle arrest at the G2/M phase. CUR induces DNA damage and activation of ATM, which phosphorylates H2A.X and CHK2 to induce cell cycle arrest at the G2/M phase by Cdc25c inhibition. CUR mediated effects may occur through a molecular mechanism dependent of ATM/Chk2/p53 signal pathway activation and confirmed our data suggesting the potent anticancer activity of CUR. Data were expressed as mean ± SD. **P < 0.01.

To demonstrate the potential role of CUR on the cell cycle distribution, the lysates were detected by flow cytometry. We found that the proportion of G2/M phase was 21.36%, 26.43%, and 31.32%, respectively, after treatment with 1, 5, and 10 μM of CUR for 24 h. For the control group, the frequency of G2/M cell cycle phase was 13.74%. The percentage of HEp-2 cell of G2/M cell cycle phase is remarkable up-regulation while that of cells in the G1-phase is decreased with the increasing concentration of CUR, as comparing with that in the control cells (Figure 3A–3D, *P* < 0.01, *n* = 3). These data indicated that CUR arrested cell cycle of HEp-2 cells in G2/M phase. To determine if CUR could mediate DNA-damage, the expression of key molecules were examined by western blot analysis. We observed an increased in phospho-H2A.X, which is the indicator of DNA double-strand breaks (DSB). Meanwhile, our present investigation indicate that CUR treatment obviously down-regulated the expression of DNA polymerase β1 (Figure 3E). Intriguingly, CUR treatment enhanced the expression of pATM without change in the protein abundance in HEp-2 cells. At the same time, we observed substantial phosphorylation of Cdc25C (Ser-216) after treatment of CUR in HEp-2 cells (Figure 3E). Exposure of HEp-2 cells with 10 μM CUR for 24 h dramatically decreased the protein abundance of Cyclin B1 compared to control cells. However, protein level of Cdc25C was similar to control group (Figure 3F). Based on the above analysis, we summarized the potential mechanism by which CUR causes cell cycle halt in HNSCCs in Figure 3G.

### CUR-induced G2/M arrest of HNSCC is associated with activation of ATM/Chk2/p53 signal transduction cascade

**Figure 4:**
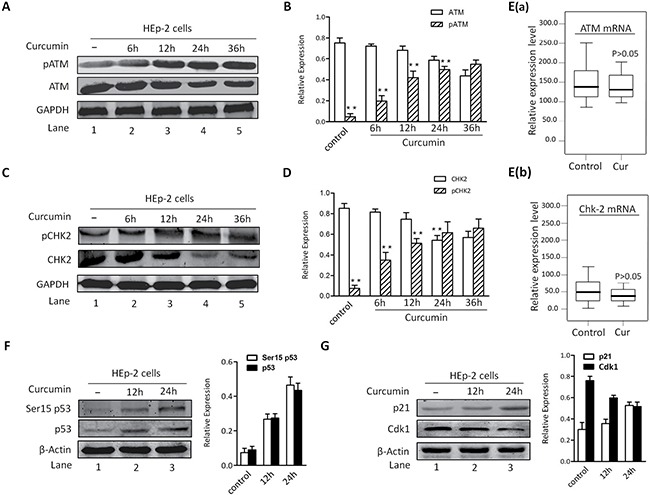
CUR-induced apoptosis is associated with activation of ATM/Chk2/p53 signal pathway (**A**–**B**) HEp-2 cells were treated with CUR (10 μM) for the indicated times and harvested. CUR triggered a significant increase in pATM levels in HEp-2 cells. (**C**–**D**) Examination of Chk2 showed a marked increase in phosphorylation in response to CUR. (**E**) The mRNA expression of ATM and Chk2 was borderline decreased by CUR pretreatment of HEp-2 cells (*p* > 0.05). (**F**–**G**) Western blot analysis using antibodies specific against p53 and p21 showed a marked increase in protein state of p53 and p21 in CUR treated HEp-2 cells. And CUR pretreatment of HEp-2 cells decreased Cdk1 expression level at 12 h, with increasing effect at 24 h. Results are mean ± SD of three independent experiments (*n* = 3; *P* < 0.005).

For the sake of more intuitive experimental results, we analyzed protein expression of ATM, pATM and Chk2 using Western blotting. After exposure to CUR, except for pATM (Figure 4A–4B), the expression level of Chk2 and ATM in HEp-2 cells were all significantly increased (Figure 4C–4D). Therefore, these results implied that CUR induced apoptosis in HEp-2 cells through an ATM/Chk2/p53-dependent pathway, at least partially. As shown in Figure 4E, the mRNA expression of ATM and Chk2 was borderline decreased by CUR pretreatment of HEp-2 cells (*p* > 0.05). Western immunoblot analysis of p53 and p21 showed a marked increase in protein state in CUR treated HEp-2 cells (Figure 4F–4G). CUR pretreatment of HEp-2 cells decreased Cdk1 protein abundance at 12 h, with enhancing effect at 24 h (Figure 4G).

### CUR inhibited tube formation and blocked migration of Human umbilical vein endothelial cells (HUVECs) *in vitro*

**Figure 5:**
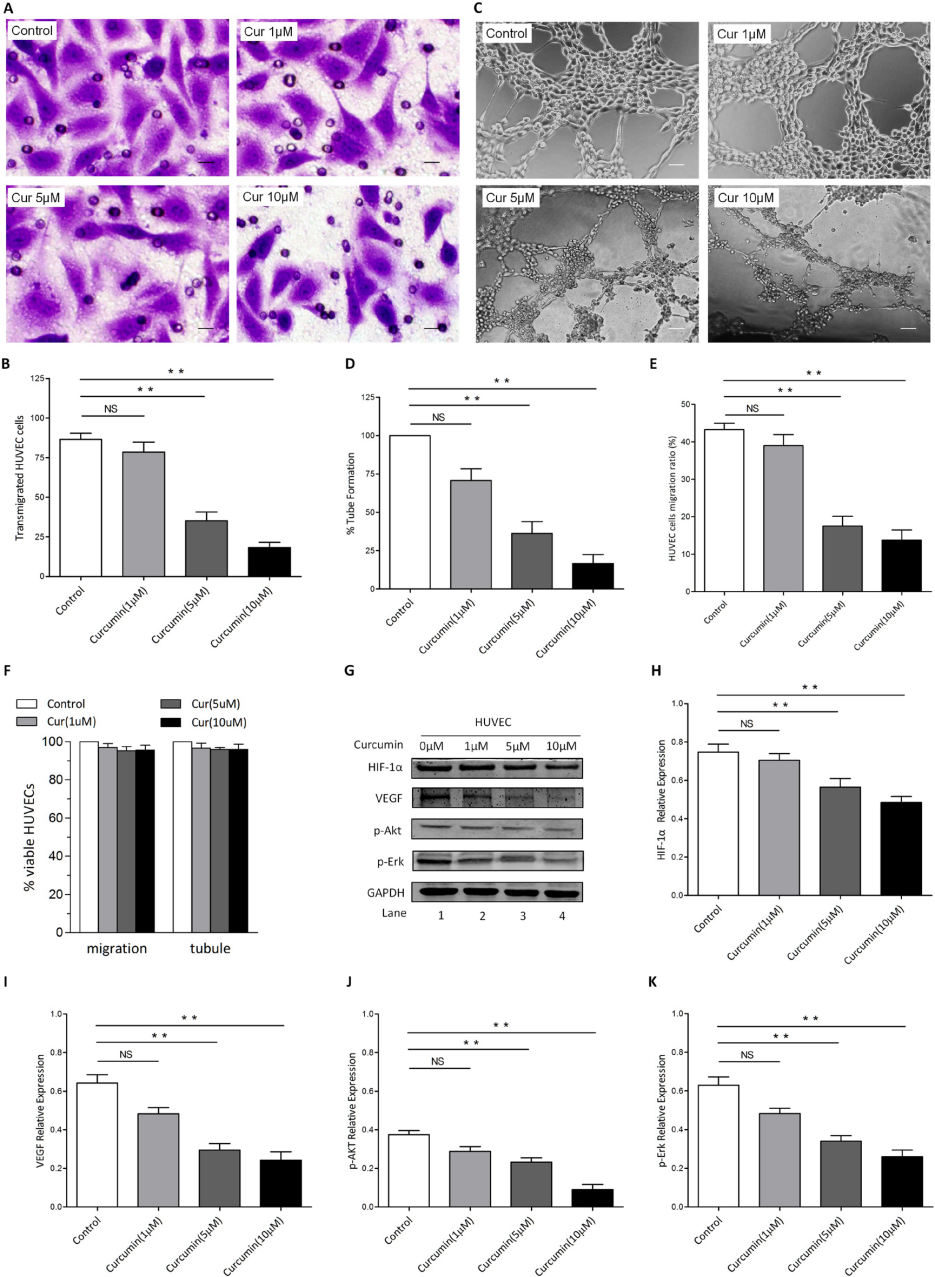
CUR blocks migration and tubule formation of HUVECs *in vitro* (**A**–**B**) CUR significantly inhibited HUVECs migration, as evidenced by a decrease in the number of crystal violet-stained cells. 10 μM CUR pretreatment of HUVECs inhibited transmigration, which was statistically significant difference to the effect of control group (scale bars, 10 μm). (**C**–**D**) HUVECs were seeded in 96-well culture plates precoated with Matrigel; and then examined for tubule formation using an inverted microscope. Tubule formation was markedly decreased in a dose-dependent manner in CUR-treated cells (scale bars, 100 μm). (**E**) Results of wound healing assay, HUVECs migration ratio after CUR treatment for 8 hours. (**F**) HUVECs were treated with or without CUR at the indicated concentrations, followed by assessment for cell viability using MTT assays. Raw data from MTT assays were normalized to the % of viable cells in control versus CUR-treated carcinoma cells. (**G**–**K**) HUVECs were treated with CUR for 24 hours, then cell lysates were collected and western blot was conducted for the indicated proteins (**P < 0.01 vs control).

In order to evaluate the anti-angiogenic and anti-migration potential of CUR and its role of action by means of ATM/Chk2/p53 signal pathway, transwell migration assay, wound healing assays and tubule formation assays were conducted to measure transmigration and tubule formation of HUVECs. Our results showed that CUR markedly inhibited HUVECs migration (Figure 5A–5B). To demonstrate the anti-angiogenic potential of CUR, we next performed Matrigel angiogenesis assay *in vitro* with HUVECs. Capillary-like tubule formation was significantly decreased in CUR treated cells in a dose-dependent fashion (Figure 5C–5D). Furthermore, scarification assay revealed that migration of HUVECs was significantly suppressed by CUR without loss of cell viability (Figure 5E–5F). A significant reduction of HIF-1α and VEGF was observed in HUVECs receiving CUR treatment (Figure 5G–5I). After treated with CUR for 24 hours, phosphorylation of Akt and Erk were observed to be decreased apparently (Figure 5J–5K).

### CUR suppressed HNSCC tumor growth in xenograft nude mice model (*In vivo*)

**Figure 6:**
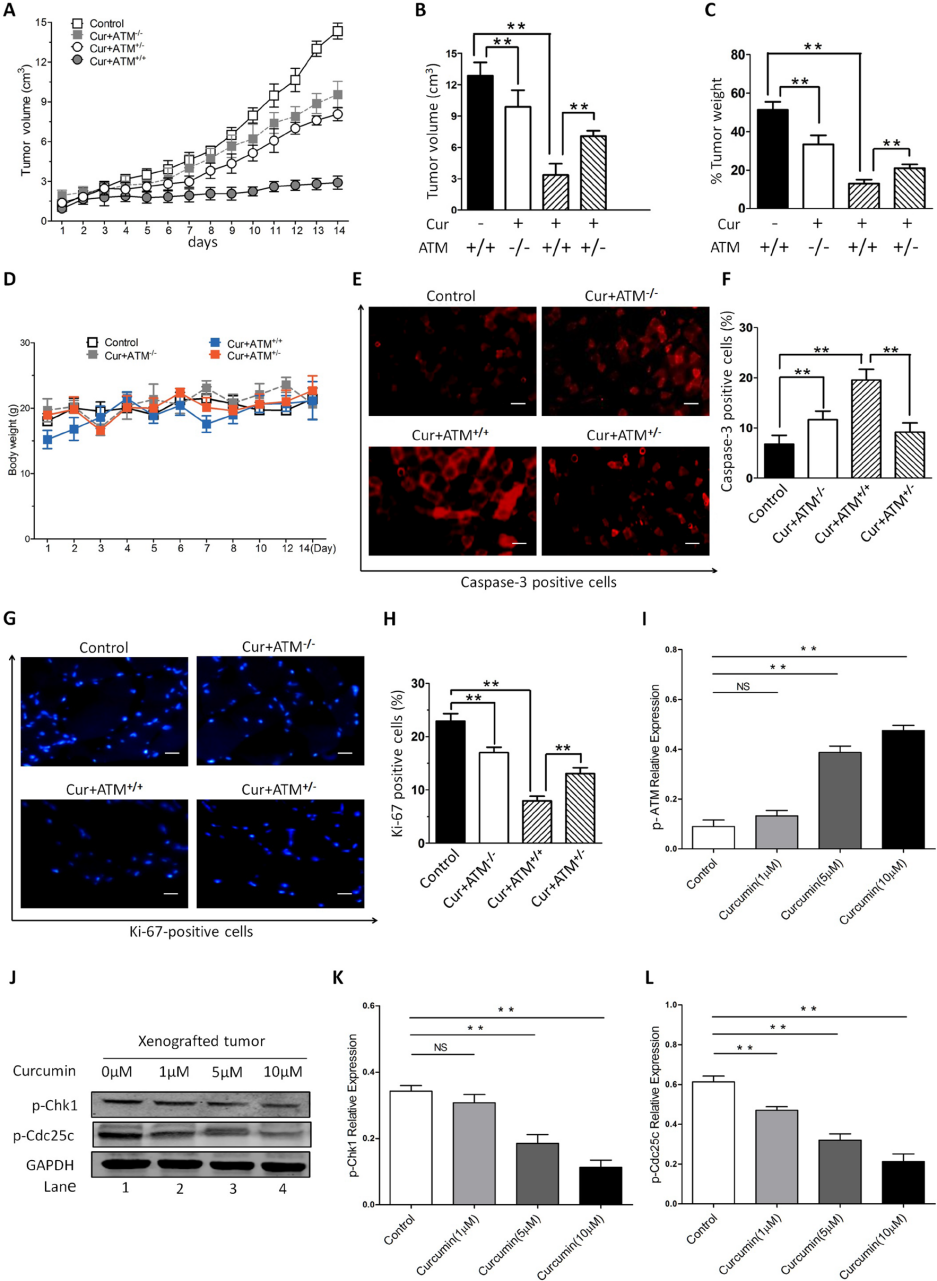
*In vivo* effects of CUR on HNSCC growth Female nude mice were subcutaneously injected into one side of ventral flanks with HEp-2 cells. The tumor volumes were measured every day from day 14 after transplantation (**A**–**B**) and weight (**C**) of tumors at day 35 posttransplantation are shown. (**D**) No significant difference of body weights was observed between different groups. (**E**–**H**) Tumors were removed from CUR-treated or control untreated mice, followed by immunofluorescence analysis with antibodies against cleaved-caspase-3 and Ki-67. (**I**) Examination of the xenografted tumor sections showed that CUR increased the number of p-ATM positive cells. (**J**–**L**) Furthermore, a significant increase in cell cycle protein Chk1 and Cdc25c was also noted in tumor sections from CUR-treated mice relative to control mice. GAPDH was used as an internal loading control. The data are expressed as mean ± SD, and significant differences from the control are indicated by **P < 0.01 (scale bars, 100 μm).

In order to further validate the interaction between CUR and ATM/Chk2/p53 signal pathway *in vivo*, xenograft nude mice model was developed to investigate underlying anti-angiogenesis activity of CUR. In our xenograft nude mice model, we observed that the nude mice tumor grew significantly slower in the CUR treated mice than that of the vector tumor model (Figure 6A–6C), which confirms the viewpoint that CUR decelerates tumor growth *in vivo*. HEp-2 cells were injected into one side of ventral flanks subcutaneously of female nude mice. The volumes of tumor were gauged every day from two weeks after transplantation. In contrast, we had observed no significant difference of body weights between different groups (Figure 6D), which demonstrates CUR has the potential against the tumor growth and shows no toxicity to tumor-bearing mice. Moreover, we found a dramatically decrease in Ki-67-positive cells in tumor sections from CUR-treated mice compared with control mice. Blockage of ATM production totally reversed CUR induced cell apoptosis in the xenografted tumor (Figure 6E–6H). In line with our *in vitro* findings, CUR significantly increased p-ATM expression in the transplanted tumor burdens (Figure 6I, Supplementary Figure 1). The xenografted tumor sections were also used to evaluate the effect of CUR on phosphorylation of Chk1 and Cdc25c, and the results showed that CUR obviously induced phosphorylation of Chk1 and Cdc25c (Figure 6J–6L), which indicated that CUR can induce the activation of ATM and target ATM mediated signaling pathways.

### CUR decreased tumor microvessel density in tumor-bearing mice

**Figure 7:**
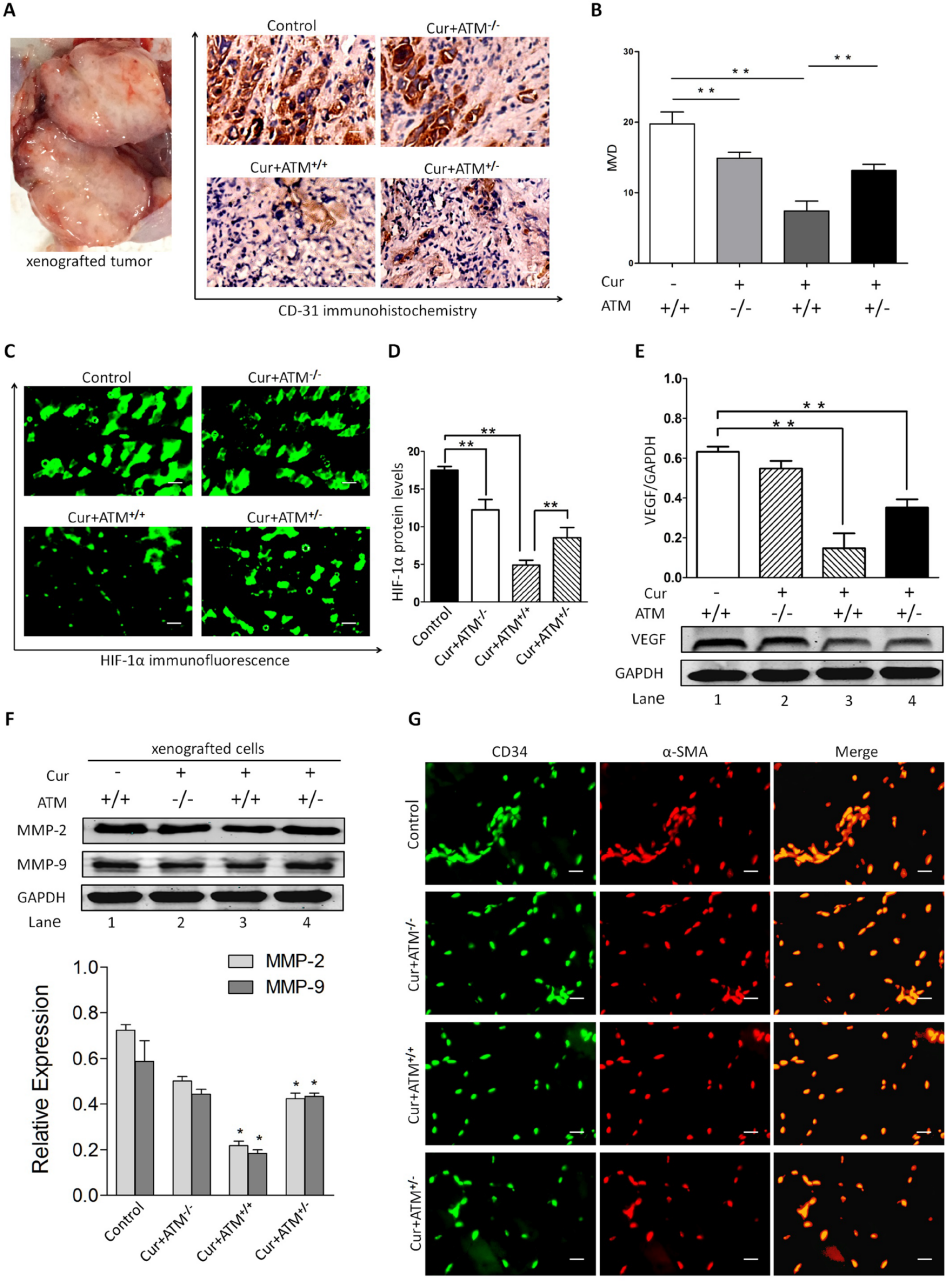
*In vivo* effects of CUR on HIF-1α expression and angiogenesis (**A**–**B**) The representative IHC staining images (scale bars, 100 μm) and the expression scores of CD31 and the numbers of MVD in HEp-2 cells originated tumors at day 35 after injection. (**C**–**D**) Immunofluorescence analysis of tumor sections showed that CUR significantly decreased HIF-1α expression compared to that of the untreated group. (**E**) A significant reduction of VEGF in tumor was observed in mice receiving CUR treatment relative to untreated mice. (**F**) Pretreatment of xenograft mice model with CUR resulted in inhibition of MMP-2 and MMP-9 production. Blockade of ATM partially reversed CUR-induced decrease in MMP-2 and MMP-9 production. These results indicate that CUR attenuates VEGF, MMP-2 and MMP-9 protein expression. (**G**) Smooth muscle actin (SMA) shows exclusive staining of the smooth muscles cells within veins and arteries and does not overlap with CD34 FITC staining which is localized in the endothelial lining of vessels. Data are mean ± SD in six mice in an independent experiment. **represents *p* < 0.01 compared with vehicle.

Further analysis of histology indicates that tumor-bearing mice with CUR treatment presented lower CD31 expression and MVDs than did vehicle infected tumors (Figure 7A–7B). Immunofluorescence analysis of tumor sections showed that CUR significantly decreased HIF-1α expression compared to that of the untreated group (Figure 7C–7D), which indicated CUR could inhibit tumor angiogenesis. Since VEGF is known to significantly induce pathological angiogenesis in tumors, the effect of CUR on VEGF and tumor angiogenesis was investigated. And we observed a dramatically decrease of VEGF in tumor sections from tumor-bearing mice receiving CUR treatment compared with untreated mice (Figure 7E, Supplementary Figure 2). And we further examined the role of CUR on MMP-9 and MMP-2 in xenograft nude mice model. As shown in Figure 7F, the expression level of MMP-9 and MMP-2 was dramatically down-regulated by CUR pretreatment of xenograft cells. Blockage of ATM production totally reversed CUR suppressed angiogenesis in the xenografted tumor (Figure 7G).

## DISCUSSION

Several studies have indicated that cells engage a complex set of events in response to DNA damage [6]. These events bring about the remove of the damage and also halt or slow down cell cycle progression until the damage has been repaired [6, 19]. The antitumor potential of CUR originates from its ability to block migration of many tumor cell lines, increase the expression of p53, p21 [20–24]; decrease the expression of Egr-1, activator protein-1 and NF-κB; suppress the expression of matrix metalloprotein-9, chemokines, LOX, cyclo-oxygen-ase-2, NOS, TNF, uPA and cyclin D1; suppress the activity of growth factor receptors; and reduce the expression of c- JNKs [25–31]. In our study, CUR blocks proliferation of HNSCC cells *in vitro*. This study reflects that the ATM/Chk2/p53 signal pathway plays a key role in curcumin-mediated cell cycle arrest in the HNSCC. HNSCC cells viability was assessed and about 75% cell death was observed after CUR treatment.

CUR has been reported to induce apoptosis and cell cycle arrest in different carcinoma cells [32–34]. In this study, we demonstrate that 5 μM CUR causes G2/M arrest in HNSCCs. We then examined the underling mechanism of DNA strand breaks induced G2/M halt by CUR in HNSCC. In addition, we found a decreased DNA polymerase-β and an up-regulation of p-H2A.X and p-ATM. Phosphorylation of H2A.X and ATM protein are indicators of DNA strand breaks and DNA polymerase beta plays an important role in DNA damage repair. And ATM is a member of PIKK that play a crucial role in the repair of DNA damage [35]. During DNA damage repair process, ATM is recruited at the DNA strand breaks sites and undergoes autophosphorylation at Ser-1981, meanwhile it triggers series of signal pathways via the phosphorylation of cell cycle proteins such as Chk2 at Thr-68 [36, 37]. Our findings indicate CUR stimulates ATM through phosphorylation at Ser-1981. The results of this study are consistent with previous findings in non-small cell lung cancer where kotomolide decreased proliferation via the activation of ATM [38]. To explore the potential role of ATM in CUR-induced G2/M halt, HNSCC cell lines were subjected to CUR in the presence or absence of KU55933, a biological inhibitor of ATM. Reduced expression of ATM obviously inhibited ATM phosphorylation at Ser-1981 induced by CUR and essentially prevented HNSCC cell lines from undergoing cell cycle arrest as well as apoptosis.

HUVECs play a role in vascular sprout and growth and often be used to evaluate anti-angiogenesis activity *in vitro* [39]. In the present study, HUVECs viability was assessed and < 5% cell death rate was found after CUR treatment, including the potential drug-induced suppression of tubule formation. We observed that CUR suppressed tubule formation of HUVECs through inhibiting HIF-1α, and it was consistent with previous studies [40, 41]. These findings indicate that CUR has the possibility to inhibit angiogenesis. In addition, several treatments have been studied in order to increase clinical therapeutic effect of anti-angiogenic therapy against hypoxic tumor chambers [42]. However, HIF-1α blockade inhibits pathological vessels as well as reduces healthy angiogenesis [43]. According to the results, decreased the expression of ATM is linked with angiogenesis and HIF-1α expression in HNSCC cells, suggestion that specific inhibitor regulating ATM pathway would be beneficial for suppressing tumor angiogenesis [44].

In summary, these experiments provide the potent evidence that CUR dramatically inhibited the proliferation of HNSCC via the activation of ATM/Chk2/p53 and suppressed angiogenesis by the inhibition of HIF-1α expression. Further study is necessary to figure out whether CUR contributes other possible molecular mechanisms through targeting ATM/Chk2/p53 pathway.

## MATERIALS AND METHODS

### Cell culture and proliferation assay

In this study, all cell lines (HEp-2, SCC-15, FaDu) were purchased from Clonetics (San Diego, CA, USA) and were maintained in Dulbecco's modified Eagle's medium containing 1% penicillin and streptomycin, supplemented with 10% fetal bovine serum (FBS). 95% humidified air was generated by flushing a 5% CO_2_ mixture into the incubator. Supernatant and cell lysates were collected at 3 days after reseeding. A total of 3 × 10^4^ cells per well were seeded onto fibronectin-coated 24-well plates, and proliferation assays were performed according to the manufacturer's instructions. After pretreatment with 5 μM/10 μM CUR for 6, 12, 24 and 36 h, or left untreated. Then, cells were re-suspended and counted. CUR was obtained from Shanghai Laboratory Animal Co Ltd (SLAC, Shanghai, China). Each condition was assessed in triplicate.

### Cell apoptosis assay

Cell apoptosis was quantified using the Annexin V/propidium iodide (PI) detection kit (Beyotime, Shanghai, China) and analyzed by flow cytometry. Cells (2 × 10^5^/well) were plated in 6-well dishes and treated with 10 μM CUR or no CUR exposed to 10 μM KU55933 or no KU55933. After treatment, collected cells were incubated in 400 μl binding buffer with 5 μl Annexin V-FITC and 5 μl PI in dark for 15 min at room temperature.

### Migration assay and wound healing assay (*in vitro*)

Transwell Insert Assays using Transwell migration chambers (BD Biosciences) with 6.5-mm-diameter polycarbonate filters ( 8 μm pore size) were utilized to measure migration as previously described [45]. In brief, cells were pretreated with CUR for 1–3 hours as indicated. Thereafter, the bottom chambers were filled with 600 μL of DMEM media containing all supplements. Cell lines (3 × 10^4^ per well) were seeded in top chambers in 100 μL DMEM media without serum. HUVECs were seeded into six-well plates to reach a confluence, which was wounded with a yellow pipette tip. The plates were incubated as above and photographed at 24 h. The migrated cells were quantified by manual counting and five randomly chosen fields were analyzed for each well.

### Capillary-like tubule structure formation assay (*in vitro*)

*In vitro* angiogenesis was assessed by Matrigel capillary-like tubule structure formation assay as described previously [46]. Matrigel-Matrix (BD Biosciences, Franklin Lakes, New Jersey, USA) was pipetted into pre-chilled 96-well plates (50 μL matrigel per well) and polymerized for 45 min at 37°C. HUVECs were seeded onto the solidified gel, and the endothelial tubes were counted under photomicroscope. Images were acquired under a fluorescent microscope (IX-71; Olympus, Tokyo, Japan) with 12.8 M pixel recording digital color cooled camera (DP72; Olympus). Capillary tubule branch points were counted in six randomly selected fields per well, and used as an index for tubule formation.

### Xenograft tumor model (*in vivo*)

Animal care and experiments were performed in strict accordance with the “Guide for the Care and Use of Laboratory Animals” and the “Principles for the Utilization and Care of Vertebrate Animals” and were approved by the Experimental Animal Ethical Committee at Shanghai Jiao Tong University School of Medicine. All animal experiments were housed four per cage on a 12:12 light: dark cycle with free access to food and water. The xenograft tumor model was performed as previously described [47]. ATM-deficient mice and female athymic nude (nu/nu) mice (3–4 weeks old) were purchased from Shanghai Laboratory Animal Co Ltd (SLAC, Shanghai, China). The generation and genotyping of the ATM^−/−^ and ATM^+/−^ mice have been previously described [48]. The mice were bred and maintained under specific pathogen-free conditions in the Animal Facility, Shanghai Jiaotong University. HEp-2 (5 × 10^6^) cells were inoculated subcutaneously into the right flank of nude mice. The volume of the implanted tumor was measured at every day with a vernier caliper, using the formula: V = L × W^2^/2; When tumors were measurable approximately one week after HEp-2 cells injection, mice were treated orally with vehicle alone (20% PEG400 deionized water) or CUR (200 mg/kg) for 4 weeks on a seven consecutive days/week schedule. The mice were sacrificed and the tumors were weighed 5 weeks after inoculation.

### Quantitative real-time PCR of mRNAs

The expression of mRNAs was assayed using TaqMan mRNA reverse transcription assays (Applied Biosystems) following the manufacturer's instructions as described previously [49]. The primers for ATM, Chk2 and GAPDH were used as showing in Table 1. The reaction conditions indicated in the manufacturer's manual were used and the reaction mixtures were incubated at 50°C for 2 min, 95°C for 10 min, followed by 40 cycles of 95°C for 15 sec and 60°C for 1 min. The ΔΔCt method for relative quantization was used to determine mRNA expression levels. The ΔCt value was calculated by subtracting the Ct of GAPDH from the Ct of the mRNA of interest. The ΔΔCt value was calculated by subtracting the ΔCt of the reference sample from the ΔCt of each sample. The fold-change was determined as 2^−ΔΔCt^.

**Table 1:** PCR primers used to amplify the mRNA

Gene	Forward primer (5′–3′)	Reverse primer (5′–3′)
*ATM*	ATCGCAGAGCGCCTCCATGTC	GAAGAACATGATCTGTGGGTG
*Chk2*	GTCATCTCAAGAAGAGGACT	GAGCTGTGGATTCATTTTCC
*GAPDH*	GGGAAACTGTGGCGTGAT	AAAGGTGGAGGAGTGGGT

### Western blotting and protein quantification assay

Western blotting analyses were performed as described previously [50] using antibodies against specific protein. GAPDH was used as a loading control. Densitometry of protein bands was acquired using an EC gel documentation system (Alpha Innotec, Kasendorf, Germany), and bands were analysed using the spot densitometry analysis tool (Alpha Ease FC software, version, 4.1.0).

### Immunohistochemistry and immunofluorescence antibody assay

Cancer tissue samples were processed for immunohistochemistry (IH) and immunofluorescence (IF) with activated caspase-3 (AC-3) and Ki-67 antibodies for the detection of apoptosis. Angiogenesis was assessed using the IHC staining of anti-CD31 antibody for nude mice cancerous tissues. Monolayers were rinsed once in phosphate-buffered saline (PBS), fixed with cold methanol for 30 min and blocked with 5% bovine serum albumin in PBS with Ca^2+^ and Mg^2+^ for 60 min. After further incubation for 15 min at 37°C, the fluorescence was measured using a fluorescence microscope (Olympus BX-40) and a Leica DFC 300FX camera. Analyses were performed by a single blinded researcher [50]. The stained slides were scored by two investigators according to the value of IRS systems.

### Statistical analysis

All the statistical analyses were performed by the statistical package for social science (SPSS) (v. 13) (SPSS Institute). Statistical significance of differences observed in drug-treated versus control cultures was determined by the Student's *t* test. Tumor volume in mice was measured using the GraphPad prism (GraphPad Software, SanDiego, CA, USA). The *p* values for comparison between line-linked groups were obtained by Student's *t* test. All statistical tests were two-sided, and *P* < 0.05 was considered to be statistically significant.

## SUPPLEMENTARY MATERIALS FIGURES


