# Supplementary material for: Curcumin induces G2/M cell cycle arrest and apoptosis of head and neck squamous cell carcinoma in vitro and in vivo through ATM/Chk2/p53-dependent pathway
Source: Oncotarget. 2017 Apr 13;8(31):50747–60. doi: 10.18632/oncotarget.17096 (PMC5584201; doi:10.18632/oncotarget.17096)
Supplement: Supplementary file 1 [file oncotarget-08-50747-s001.pdf]

Curcumin induces G2/M cell cycle arrest and apoptosis of head and neck squamous cell carcinoma *in vitro* and *in vivo* through ATM/Chk2/p53-dependent pathway

Supplementary Materials

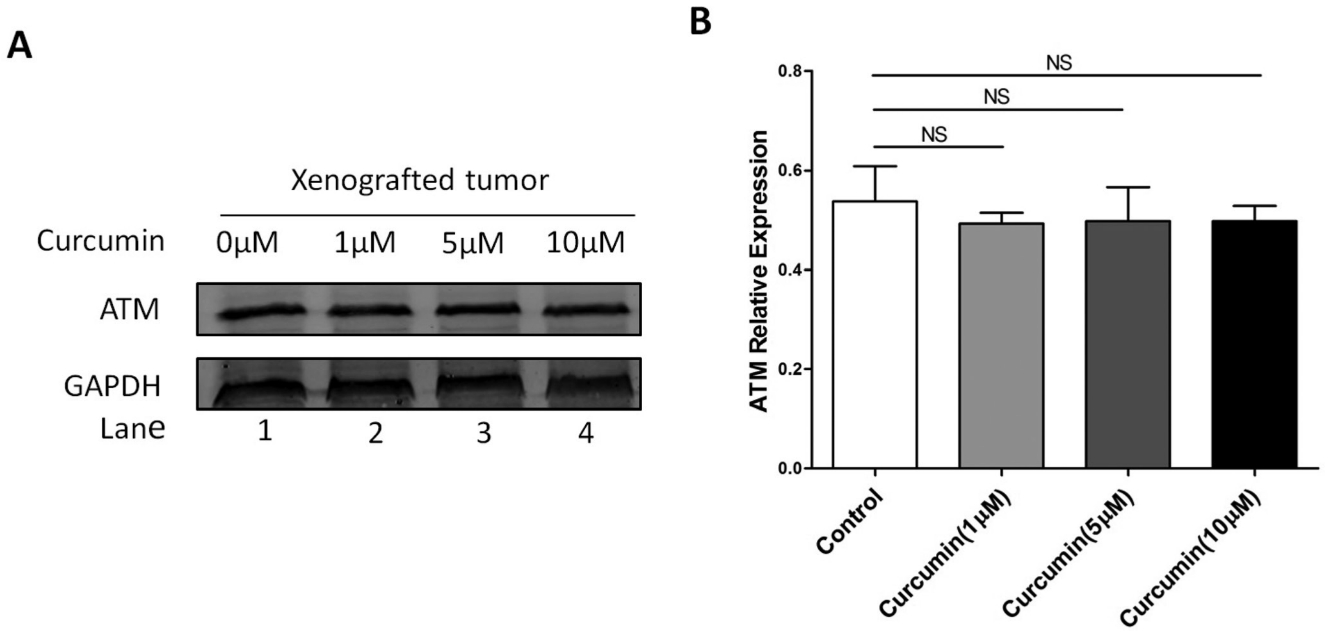

Supplementary Figure 1: CUR-induced apoptosis is associated with activation of ATM signal pathway. (**A–B**) Female nude mice were subcutaneously injected into one side of ventral flanks with HEp-2 cells. The expression of ATM was borderline decreased by CUR pretreatment ($p > 0.05$). The data are expressed as mean ± SD, and significant differences from the control are indicated by $^{**}p < 0.01$.

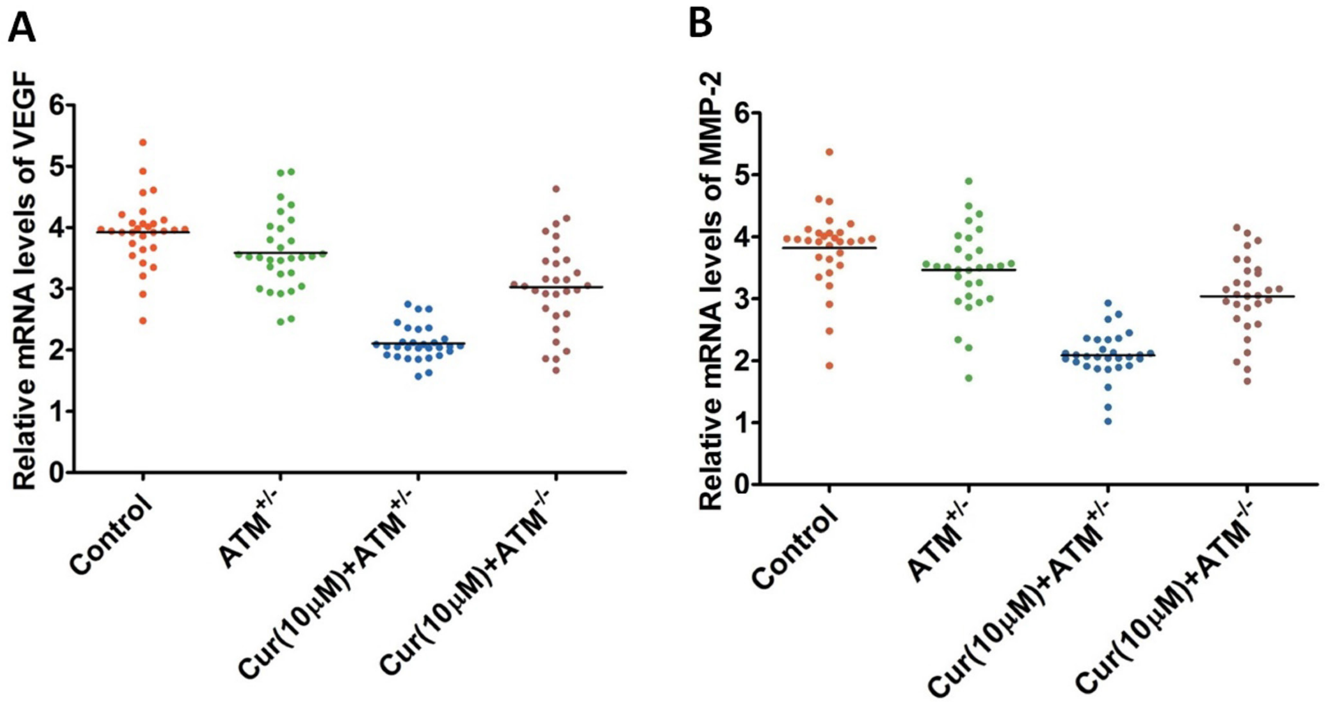

Supplementary Figure 2: Pretreatment of xenograft mice model with curcumin resulted in inhibition of VEGF and MMP-2 production. (**A**–**B**) Semi-quantitative reverse transcription–PCR of VEGF and MMP-2 in xenograft cells demonstrates that MMP-2 and VEGF mRNA was down-regulated expression. Blockade of ATM partially inhibited curcumin-induced decrease in VEGF and MMP-2 production. These results indicate that curcumin attenuates both VEGF and MMP-2 protein and mRNA expression. GAPDH was used as an internal loading control. The data are expressed as mean ± SD, and significant differences from the control are indicated by $^{**}p < 0.01$.
